# Voltage-Activated Ion Channels in Non-excitable Cells—A Viewpoint Regarding Their Physiological Justification

**DOI:** 10.3389/fphys.2018.00450

**Published:** 2018-04-27

**Authors:** Lars Kaestner, Xijia Wang, Laura Hertz, Ingolf Bernhardt

**Affiliations:** ^1^Theoretical Medicine and Biosciences, Saarland University, Homburg, Germany; ^2^Experimental Physics, Saarland University, Saarbrücken, Germany; ^3^Laboratory of Biophysics, Saarland University, Saarbrücken, Germany; ^4^Medical Faculty, Institute for Molecular Cell Biology, Saarland University, Homburg, Germany

**Keywords:** red blood cells, Ca_V_2.1, Piezo1, hSK4 (KCNN4), Calcium signaling, Gardos-channelopathy

It is a well-known fact that voltage-activated ion channels are expressed in non-excitable cells (Jagannathan et al., 2002; Piskorowski et al., 2008; Sontheimer, 2008). However, their putative physiological functions and the regulation of their activity in non-excitable cells are controversial topics (Stokes et al., 2004; Badou et al., 2013). This also holds true for red blood cells (RBCs).

In the context of investigating ion transport across the membrane of RBCs, especially in low ionic strength media, the existence of ion transport dependent on the membrane potential was reported for the first time approximately 50 years ago (Donlon and Rothstein, 1969). In an investigation based on comparative physiology, it became evident that the low ionic strength-induced cation permeability in RBCs is not due to electrodiffusion but due to a transport protein-based process (Halperin et al., 1990; Bernhardt et al., 1991). Later, in addition to the K^+^(Na^+^)/H^+^ exchanger (Richter et al., 1997; Kummerow et al., 2000), the existence of a voltage-activated non-selective cation channel was functionally demonstrated utilizing the patch-clamp technique (Christophersen and Bennekou, 1991; Kaestner et al., 1999; Rodighiero et al., 2004). At present, the molecular identity of this particular channel remains unknown (Kaestner, 2011; Bouyer et al., 2012), and it has alternatively been proposed to reflect a conductance state of the voltage-dependent anion channel (VDAC) (Bouyer et al., 2011). On the other hand, evidence for the existence of a number of voltage-activated Ca^2+^ channels that are abundant in RBCs has been reported (Pinet et al., 2002; Romero et al., 2006), and the most convincing evidence is for Ca_V_2.1, based on molecular biology data (Western blot) (Andrews et al., 2002) and, presumably, Ca_V_2.1-specific pharmacological interactions (ω-agatoxinTK) (Andrews et al., 2002; Wagner-Britz et al., 2013). Nevertheless, so far, we and others have failed to obtain direct functional evidence for the existence of Ca_V_2.1 or other voltage-activated Ca^2+^ channels in RBCs by patch-clamp techniques.

Although RBCs are undoubtedly non-excitable cells, sudden changes in membrane potential occur, when increased cation permeability is induced. This is the case because the resting membrane potential is determined by Cl^−^ conductance (Hunter, 1977; Lassen et al., 1978). For example, when the Gardos channel (Gardos, 1958; Hoffman et al., 2003) is activated, the resting membrane potential changes from approximately −10 to −90 mV (Tiffert et al., 2003). The physiological function of the Gardos channel remained elusive for decades, until it was discovered that it is a major component of the suicidal process of RBCs (Kaestner and Bernhardt, 2002; Lang et al., 2003; Bogdanova et al., 2013) triggered by Ca^2+^ entry (Yang et al., 2000; Kaestner et al., 2004), resulting in cell shrinkage (Begenisich et al., 2004; Lew et al., 2005), and phosphatidylserine exposure (Chung et al., 2007; Nguyen et al., 2011). Only very recently was the interplay between the mechanosensitive channel Piezo1 and the Gardos channel established (Faucherre et al., 2013; Cahalan et al., 2015; Danielczok et al., 2017a), showing a Ca^2+^-mediated response when RBCs pass through constrictions such as small capillaries.

Since patch-clamp protocols for Ca_V_2.1 in RBCs are lacking, imaging approaches based on the Ca^2+^ fluorophore Fluo-4 are the method of choice (Minetti et al., 2013). The sudden change in membrane potential following Gardos channel activation suggests that there is a link between Gardos channel activity and voltage-activated channels. Such an interplay was already demonstrated in a recent publication showing an 80% reduction in lysophosphatidic acid (LPA)-induced Ca^2+^ entry by the Gardos channel blocker charybdotoxin (Figure 4A in Wesseling et al., 2016). It would be nice to show Ca_V_2.1 activity in direct response to Gardos channel activation. This is challenging because the activation stimulus for the Gardos channel (an increase in intracellular Ca^2+^) is the same parameter used to measure Ca_V_2.1 activity. However, Gardos channel activity is increased in patients carrying a mutation that affects the calmodulin-binding site (R352H) (Rapetti-Mauss et al., 2015; Fermo et al., 2017). This could be used as a model to investigate the putative interplay between the Gardos channel and Ca_V_2.1. One would expect an increase in Ca_V_2.1 activity due to increased Gardos channel activity. Consequently, intracellular Ca^2+^ levels should be elevated in the RBCs of these patients, which indeed is the case (Figure 6 in Fermo et al., 2017). The finding that only a subpopulation of cells showed increased Ca^2+^ levels (Fermo et al., 2017) can probably be explained by the highly heterogeneous distribution of the participating channels, which is well-established for the Gardos channel (Grygorczyk et al., 1984; Lew et al., 2005) but is also likely to apply to other channels in RBCs (Kaestner, 2015).

Nevertheless, we are still left with one peculiarity: According to previous investigations, Ca_V_2.1 activation is induced by depolarization (Catterall, 2011), not by hyperpolarization, which is the outcome of Gardos channel activity. However, hyperpolarization is a requirement to switch Ca_V_2.1 channels from the inactivated state to the closed state, which is a prerequisite to subsequently transition to the open state (Catterall, 2000) (Figure 1A). Closing of the Gardos channels after their initial activation could well provide the necessary conditions for subsequent depolarisation to activate Ca_V_2.1. Since the hypothetical switching behavior of the Gardos channel would be crucial for the activation of Ca_V_2.1, we would like to discuss this aspect in more detail. We envision three principle modes by which this switching could occur:

- Because channel activity is a stochastic event and because the number of Gardos channels per RBC is rather low (in the single digit numbers; Grygorczyk et al., 1984; Wolff et al., 1988), depolarisation could be the result of stochastic Gardos channel closures. This hypothesis is supported by the rather sparse whole cell patch-clamp recordings of Gardos channel activity in human RBCs (Qadri et al., 2011; Kucherenko et al., 2012, 2013; Fermo et al., 2017). Whole cell current traces do not show a smooth appearance but rather a flickering pattern similar to that observed with single channel recordings, especially at higher (positive and negative) membrane potentials.
- When looking at Gardos channel-induced changes in the membrane potential of cell populations, a gradual Ca^2+^ concentration-dependent effect can be seen (Baunbaek and Bennekou, 2008), i.e., the hyperpolarisation observed in RBC suspensions is a gradual Ca^2+^ concentration-dependent effect. However, the abovementioned study (Baunbaek and Bennekou, 2008) as well as another report (Seear and Lew, 2011) showed that the activation of the Gardos channel at the cellular level is an all-or-none response. This means that the gradual change in membrane potential would be the result of the summation of cells with open or closed Gardos channels. Taking into consideration that the Ca^2+^ pump (Schatzmann, 1973) continuously operates in response to any increase in intracellular Ca^2+^ levels, one would imagine that the state of the Gardos channels is exclusively modulated by variations in intracellular Ca^2+^ concentrations. Hence, the switching behavior of the Gardos channel would be the direct consequence of continuous variations in RBC intracellular Ca^2+^ concentrations.
- Localized interactions between the Gardos channel and Ca_V_2.1 in RBCs could occur in lipid rafts or nanodomains, as is the case with closely related ion transporters in other cell types, for example, within the fuzzy space or dyadic cleft in myocytes (Lines et al., 2006). Although RBCs do not possess membrane-constricted subspaces, there are indications for functional compartments in the immediate vicinity of the plasma membrane (Chu et al., 2012). Colocalization of ion channels is common in excitable cells (Rasband and Shrager, 2000; Bers, 2002). For RBCs, it is still unknown if the different ion channels colocalize or cluster to allow their interaction in nanodomains. However, in support of this idea is the observation that local activation of mechanosensitive channels (most likely Piezo 1) by patch-clamp micropipettes resulted in local activation (single-channel recordings) of the Gardos channel (Dyrda et al., 2010).

**Figure 1:**
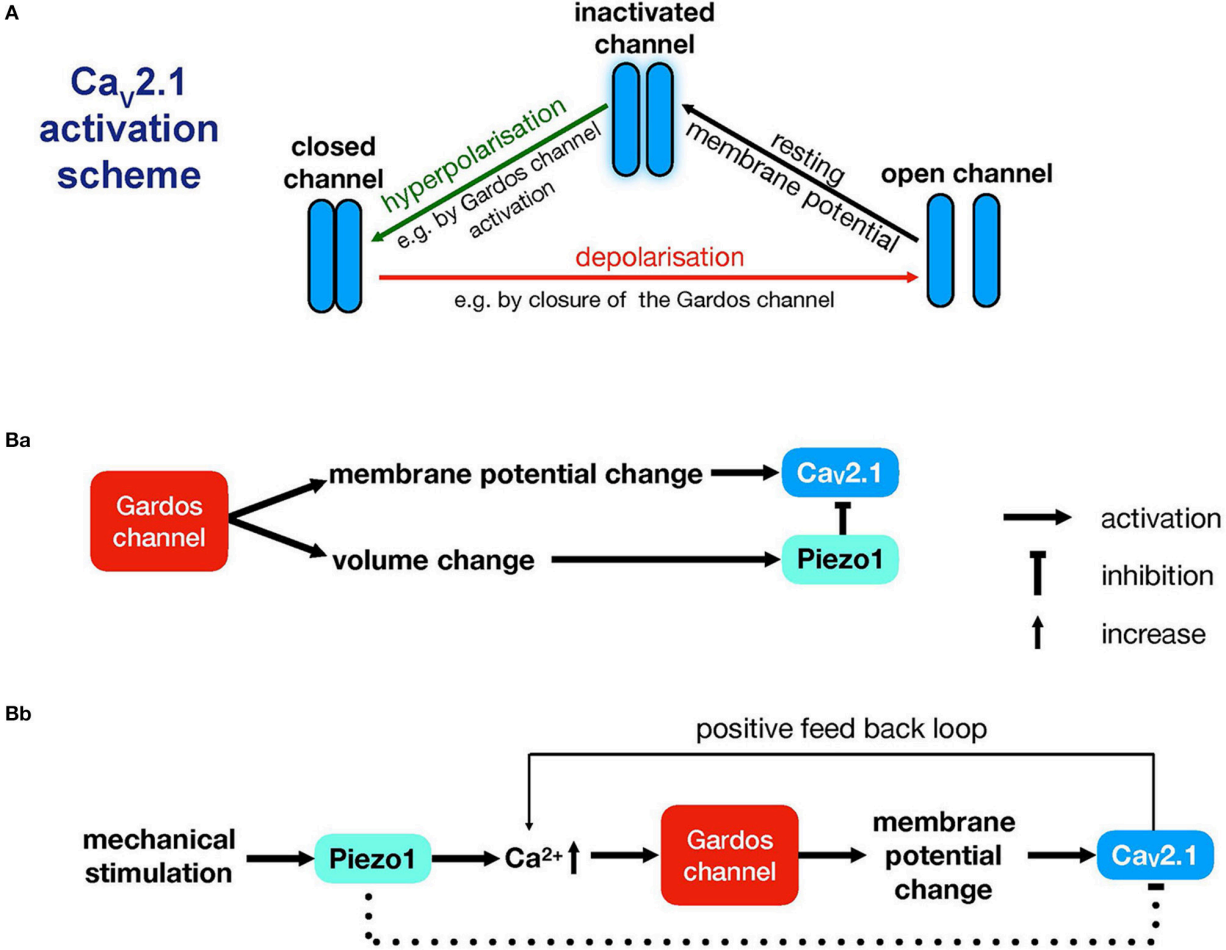
Interplay between Ca_V_2.1, Piezo1 and the Gardos channel. **(A)** Activation scheme for the Ca_V_2.1 channel modulated by underlying Gardos channel activity. **(B)** Proposed interactions between Ca_V_2.1, the Gardos channel and Piezo1. **(Ba)** illustrates a mechanism that is compatible with the measurements presented in Wesseling et al. (2016) and that explains the pathophysiological events seen in Gardos channelopathy (Fermo et al., 2017). **(Bb)** illustrates a sequence of events that is more likely to be relevant to physiological mechanical stimulation conditions in the circulation (Faucherre et al., 2013; Cahalan et al., 2015; Danielczok et al., 2017a). Whether Piezo1 would exert an inhibitory effect in this scenario is an open question, and therefore, this is depicted with a dashed line. For more details, see the main text.

Although the previous three lines of argumentation are to some extent speculative and although we are unable to favor one over the others, we believe it is worthwhile to share our thoughts with both the RBC and Ca_V_ channel research communities in this opinion article. The concerted activity of channels is essential in numerous physiological mechanisms, such as the generation of action potentials in neurons (Rojas et al., 1970), excitation-contraction coupling in the heart (Bers, 2002), and during the formation of the immunological synapse (Quintana et al., 2006). In RBCs, there is evidence that the Gardos channel is activated in response to the opening of Piezo1 (Dyrda et al., 2010; Danielczok et al., 2017a), but the inverse process may also occur; activation of the Gardos channel may induce Piezo1 activity. Since Gardos channel activation is supposed to be associated with RBC volume changes, this effect is likely to activate the mechanosensitive channel Piezo1.

To imagine what may happen when Piezo1 is activated, we need to consider the membrane potential. Activation of Piezo1, which is a non-selective cation channel, would lead to a disruption of the hyperpolarisation induced by the Gardos channel, thus preventing voltage activation of Ca_V_2.1. Therefore, if Piezo1 is closed, Ca_V_2.1 activation would be facilitated, resulting in increased intracellular Ca^2+^ compared to control conditions. We propose two scenarios explaining the interplay between the Gardos channel, Ca_V_2.1 and Piezo1 (Figure 1B).

The first scenario takes into account the RBCs of patients carrying the R352H mutation (Rapetti-Mauss et al., 2015; Fermo et al., 2017) or the V282M/E mutation (Andolfo et al., 2015; Glogowska et al., 2015; Rapetti-Mauss et al., 2015). The RBCs of these patients show increased baseline Gardos channel activity, which is schematically sketched in Figure 1Ba. This sequence of events can be initiated by the abovementioned mutations or by an increase in intracellular Ca^2+^ independent of mechanical stress, e.g., by NMDA receptor activity (Makhro et al., 2013), TRPC channel openings (Danielczok et al., 2017b), or VDAC activity (Bouyer et al., 2011).

The second scenario envisions an alternative, independent sequence of events. Piezo1 may indirectly modulate Ca_V_2.1 activity, as outlined in Figure 1Bb. If Piezo1 is the source of the increase in intracellular Ca^2+^, then subsequent Gardos channel activity would induce the opening of Ca_V_2.1 channels, while Piezo1 channels might still be in an inactivated state. It is likely that Piezo1 channels would remain inactive. After mechanical stimulation, inactivation occurs within 100 ms (Wu et al., 2017), and channel reopening would require a new (repetitive; not lasting) mechanical stimulation (Lewis et al., 2017). Whether the volume change induced by mechanical stress is sufficient for repetitive activation remains unclear, and therefore, the inhibitory effect by Piezo1 is indicated by a dashed line in Figure 1Bb. This mechanism (Figure 1Bb) might explain the long-lasting Ca^2+^ signal seen after mechanical stimulation and reported in this Research Topic (Danielczok et al., 2017a).

In summary, here, we propose several reasonable mechanisms (Figure 1) to explain how voltage-activated (Ca^2+^) channels could fulfill a physiological function in non-excitable RBCs. We hope to initiate a discussion on this topic and to encourage further investigations beyond the content of this paper.

## Author contributions

LK designed the experimental approach. XW and LH performed and analyzed the experiments. IB and LK supervised the experiments. LK wrote the manuscript. LH and IB revised the manuscript.

### Conflict of interest statement

The authors declare that the research was conducted in the absence of any commercial or financial relationships that could be construed as a potential conflict of interest.
